# Compartmentalized organ-on-a-chip structure for spatiotemporal control of oxygen microenvironments

**DOI:** 10.1007/s10544-022-00634-y

**Published:** 2022-10-21

**Authors:** Kaisa Tornberg, Hannu Välimäki, Silmu Valaskivi, Antti-Juhana Mäki, Matias Jokinen, Joose Kreutzer, Pasi Kallio

**Affiliations:** Micro- and Nanosystems Research Group, Faculty of Medicine and Health Technology, Tampere University, 33720 Tampere, Finland

**Keywords:** Organ-on-a-chip, Hypoxia, Gas control, Gas microenvironment, Oxygen concentration, Microfluidic

## Abstract

Hypoxia is a condition where tissue oxygen levels fall below normal levels. In locally induced hypoxia due to blood vessel blockage, oxygen delivery becomes compromised. The site where blood flow is diminished the most forms a zero-oxygen core, and different oxygenation zones form around this core with varying oxygen concentrations. Naturally, these differing oxygen microenvironments drive cells to respond according to their oxygenation status. To study these cellular processes in laboratory settings, the cellular gas microenvironments should be controlled rapidly and precisely. In this study, we propose an organ-on-a-chip device that provides control over the oxygen environments in three separate compartments as well as the possibility of rapidly changing the corresponding oxygen concentrations. The proposed device includes a microfluidic channel structure with three separate arrays of narrow microchannels that guide gas mixtures with desired oxygen concentrations to diffuse through a thin gas-permeable membrane into cell culture areas. The proposed microfluidic channel structure is characterized using a 2D ratiometric oxygen imaging system, and the measurements confirm that the oxygen concentrations at the cell culture surface can be modulated in a few minutes. The structure is capable of creating hypoxic oxygen tension, and distinct oxygen environments can be generated simultaneously in the three compartments. By combining the microfluidic channel structure with an open-well coculture device, multicellular cultures can be established together with compartmentalized oxygen environment modulation. We demonstrate that the proposed compartmentalized organ-on-a-chip structure is suitable for cell culture.

**Supplementary Information:**

The online version contains supplementary material available at 10.1007/s10544-022-00634-y.

## Introduction

Oxygen has a crucial role in the function and signaling of cells. Supply and consumption define the oxygen availability of cells in tissues, and when oxygen supply via the vasculature falls below the normal level, tissues experience hypoxia (Carreau et al. 2011). Low oxygen concentrations in tissues are linked to pathologies such as brain stroke, heart infarction and cancer (Michiels 2004). In regard to developing new therapies for stroke and heart infarction, even though a massive amount of research effort has been invested in animal models of these pathologies, the results suffer from insufficient translation into clinically relevant results (Chen and Vunjak-Novakovic 2018; Fluri et al. 2015). Therefore, there is a need for technologies to model low oxygen environments in human cells to study the molecular mechanisms behind these pathologies and to advance new therapies.

When an artery is blocked in the brain, the oxygen concentration within the core of this tissue section declines rapidly toward zero, resulting in anoxia. Moreover, the area around the occlusion becomes incompletely perfused and further compromises the oxygen supply to cells, creating hypoxia (Rink and Khanna 2011). Cells close to the functioning vessels experience a normal oxygen environment and normoxia. Cells in these distinctly different oxygen environments can be separated by different biomarkers and have inherently different prospects for recovery after the blood flow has been restored to the tissue site (Hossmann 2012). Furthermore, the restoration of blood flow to hypoxic tissue introduces an additional reoxygenation injury to the tissue site, which is a phenomenon occurring in not only the brain but also the kidneys and the heart (Nour et al. 2013).

Microsystems and microfluidic technologies provide means to control the microenvironments of cell cultures with high spatiotemporal precision. As in tissues, the oxygen availability in microfluidic devices is defined by supply and consumption. In addition, the material choices can be important, especially in terms of oxygen permeability (Oomen et al. 2016). In applications where cells are cultured in perfused microchannels, oxygen availability can be controlled by adjusting the flow rate of fresh medium so that the supply and consumption form a dynamic equilibrium and result in oxygen values that resemble the oxygen values found *in vivo* (Domansky et al. 2010). However, to have more control over cellular oxygen environments, without introducing additional disturbances, such as shear stress, oxygen supply structures can be separated from culture compartments with gas-permeable membranes or walls, most often made of polydimethylsiloxane (PDMS). In addition to being a popular material for fabricating organ-on-chip structures, PDMS has a relatively high oxygen permeability (Brennan et al. 2014). Oppegard and coworkers have shown that by adding gas channels to one side of a PDMS membrane, the oxygen concentration in the other side in the immediate proximity of this membrane can be changed in under a minute (Oppegard et al. 2010, 2009). This combination of gas channels and PDMS membranes has been utilized further to induce a homogenous oxygen environment for the cells (Brennan et al. 2015; Di Caprio et al. 2015).

With microfluidic technology, rapid changes in the oxygen microenvironments can be introduced. Rapid changes are not required for every cell model, but in ischemic pathologies where the oxygen supply to an organ is suddenly disrupted, quick changes and short periods of hypoxia are biologically relevant. For example, microfluidic devices modeling brain stroke and cardiac ischemia can induce hypoxic conditions that last only minutes, followed by a reperfusion period, restoring the normoxic state (Martewicz et al. 2012; Mauleon et al. 2012). Most often, microfluidic devices induce a homogenous oxygen environment that changes accordingly using only one type of cell (Ehsan and George 2015; Funamoto et al. 2017, 2012; Martewicz et al. 2012). The heterotypic nature of local hypoxia is often overlooked, and the technological solutions to model this phenomenon remain limited.

Local oxygen environments are usually established by an oxygen gradient that is created across a cell culture compartment, utilizing microfluidic channels placed beneath thin PDMS membranes (Adler et al. 2010; Lo et al. 2010; Polinkovsky et al. 2009). Even though an oxygen gradient is established, extreme oxygen concentrations are difficult to achieve. If oxygen gradient is established in a single culture chamber, the exact location of the cells within the chamber corresponds to the oxygen environment defining the cell response. Most often, the analyses are image-based because once cells are detached from the culture compartment for sample analysis, the information about the spatial location and the corresponding oxygen environment of the cells is lost (Palacio-Castañeda et al. 2022). Rexius-Hall et al. resolved this by constructing two sets of oxygen supply channels beneath the cell culture compartment, inducing a hypoxic environment on the one side and a normoxia environment on the other. Upon sample collection, the cells were scraped separately from each side to study how cells respond to different oxygen environments (Rexius-Hall et al. 2014). Hypoxia responses were studied with two different cell lines at separate times, but to study the interaction of the two cell lines requires some coculture setups in addition to the oxygen control.

To include multiple cell types within the same hypoxic tissue site, ex vivo pancreatic islets and brain tissue slices have been used together with oxygen controlling devices (Lo et al. 2012; Mauleon et al. 2012). The latter study showed how to locally stimulate different parts of brain tissue slices with different oxygen concentrations. Even though these studies demonstrated the capability of creating a heterotypic hypoxic environment, even with great spatial resolution, these approaches preclude the possibility of examining the cellular responses either by cell type or by the specific oxygen environment. To achieve a greater analytical specificity within organ-on-a-chip structures, cells should be compartmentalized either by type or by the hypoxia treatment or, ideally, by both.

In this paper, we report on a device containing a microfluidic channel structure that can create distinct oxygen microenvironments in dedicated compartments. The device structure contains three separate microchannel arrays that guide gases to diffuse through a 20 µm thick membrane to defined cell culture areas. To characterize the spatiotemporal behavior of this channel structure, we utilized 2D ratiometric oxygen imaging as shown by Ungerböck et al. (Ungerböck et al. 2013) to study the surface oxygen tension modulation dynamics and spatial resolution. The suitability of the structure for cell culture was demonstrated by cell viability for up to ten days on top of the structures coated with fibronectin. The microfluidic channel structure combined with a previously published coculture device (Ristola et al. 2019) allows cellular compartmentalization by type and oxygen concentration simultaneously, thus paving the way for a multiculture platform with compartmentalized oxygen control.

## Materials and methods

### Microfluidic channel structure

**Fig. 1:**
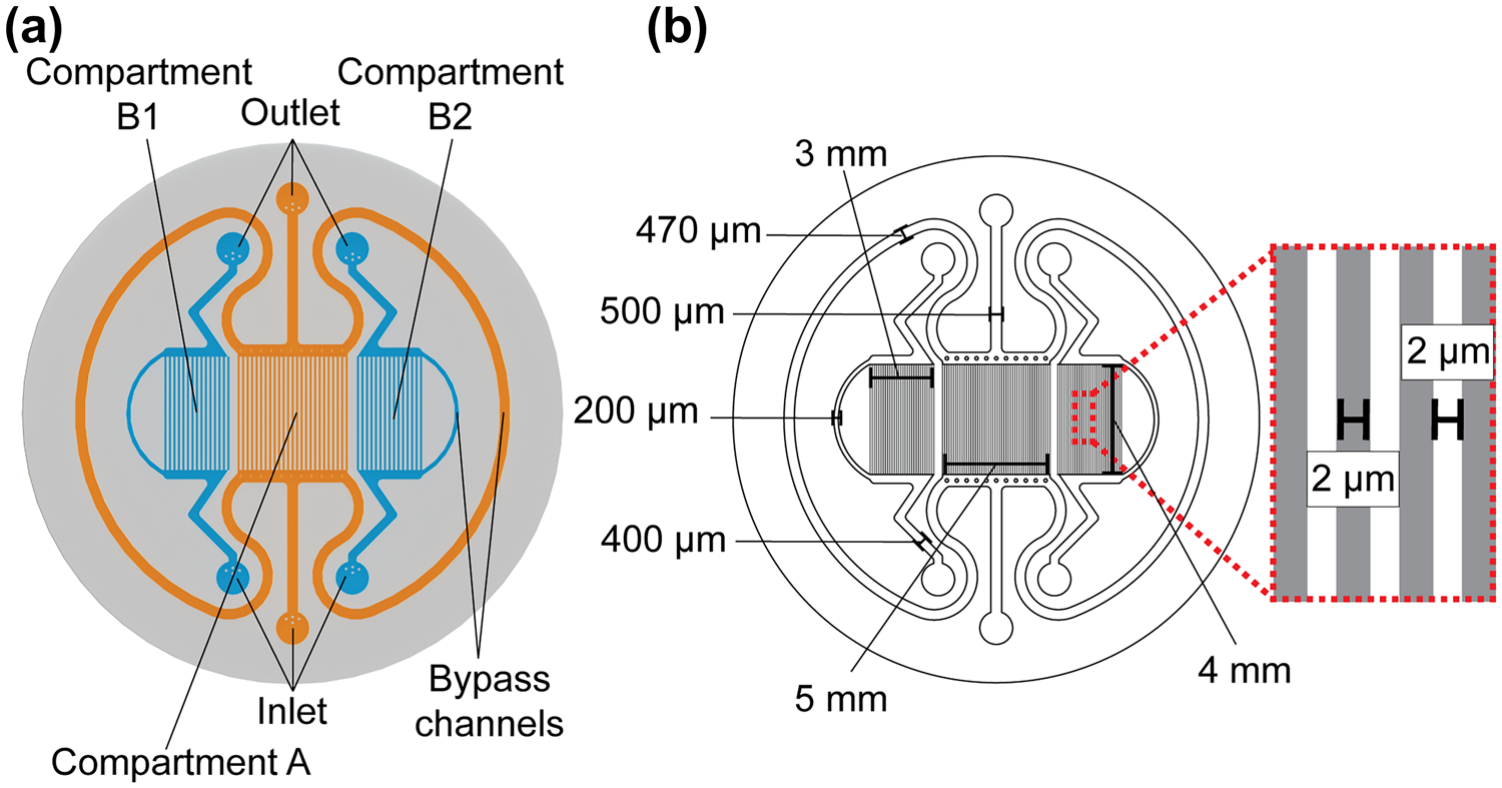
Microfluidic channel structure sealed with a 20 µm thick membrane **a** containing Compartment A and two identical Compartments B1 and B2. **b** Flow channels, including inlets, outlets and bypass channels, with a height of 100 µm guide gases to the 4 mm long microchannel array (2 µm height, 2 µm width and 2 µm spacing)

The core of the device has a microfluidic channel structure (Fig. 1a), including three separate compartments: Compartment A in the middle and Compartments B1 and B2 on the sides. Each compartment contains an array of 2 µm wide × 2 µm high microchannels with 2 µm spacing (Fig. 1b). The size of the array area is 5 mm × 4 mm in Compartment A and 3 mm × 4 mm in Compartments B1 and B2. The microfluidic channel structure was sealed using a thin (20 µm) commercially available Silpuran (SILPURAN® Film 2030 250/20, Wacker Chemie AG, Germany) membrane, which allows the gas conditions in the microfluidic channel structure to be transferred to cell culture areas via diffusion. In addition, each compartment has an individual bypass channel that reduces the pressure difference between the inlet and the outlet, thus preventing the membrane from deflecting. The height of the bypass channels and the flow channels preceding and following the microchannel arrays is 100 µm. The widths are illustrated in Fig. 1b. The oxygen environment in the cell culture area of each cell compartment can be individually controlled simply by supplying separate gases to the separate compartments of the microfluidic channel structure. For oxygen profile modulation, gas mixtures were supplied to each compartment with a flow rate of 3–5 ml/min.

### Mold and PDMS component fabrication

To make the microfluidic channel structure, a mold was fabricated using a multilayer SU-8 rapid prototyping method. The mold was fabricated using two chrome-on-glass masks, the first mask containing the 2 µm high microchannel arrays and the second one containing the 100 µm high flow channels. The computer-aided design (CAD) models prepared in SolidWorks (Dassault Systèmes, Vélizy-Villacoublay, France) were converted in AutoCAD (Autodesk, California, USA) to a format used by the direct-writer photolithography system µpg501 (Heidelberg Instruments Mikrotechnik GmbH, Heidelberg, Germany) with mask blanks (Clean Surface Technology Co., Tokyo, Japan). These masks were used together with SU-8 5 and SU-8 3050 photoresists (Micro resist technology GmbH, Berlin, Germany) spin coated on a silicon wafer producing the two layers with different heights. The SU-8 5 layer was spin-coated to a height of 2 µm where the microchannel arrays were developed. The SU-8 3050 layer was spin-coated to a height of 100 µm, and the inlet and outlet channels together with the bypass channels were developed onto this layer. Before spin-coating, the silicon wafers were treated with oxygen plasma at 30 W for two minutes with Vision 320 Mk II RIE (Advanced Vacuum, Malmö, Sweden).

To fabricate the microfluidic channel structure, the SU-8 mold was used for PDMS casting. Degassed PDMS (Sylgard 184 from Dow Corning, Midland, USA) at a ratio of 1:10 (curing agent:base elastomer) was spin coated on the SU-8 mold at a speed of 300 rpm for 30 s and cured in a 60 °C oven for 10 h, obtaining an approximately 0.2 mm thick sheet with embedded gas channels.

**Fig. 2:**
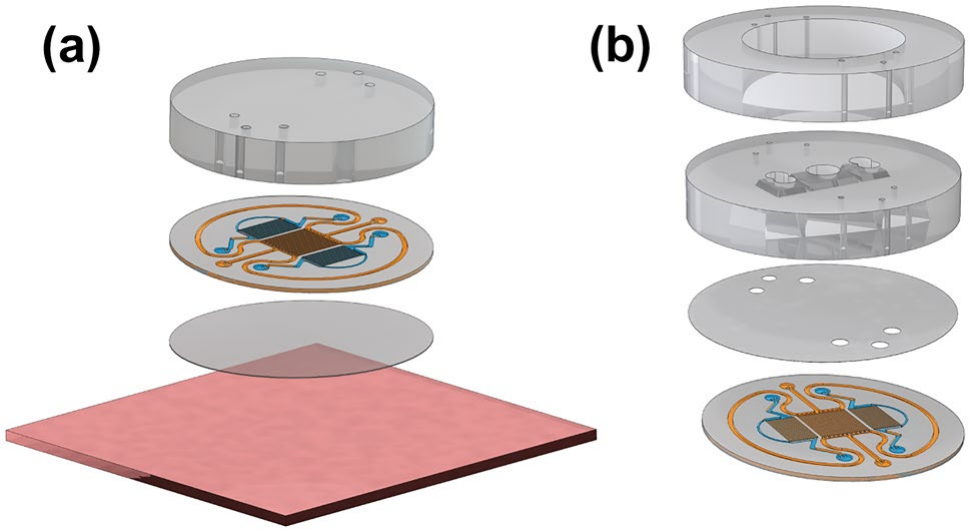
Spatiotemporal oxygen behavior characterization: **a** the microfluidic channel structure sealed with the 20 µm thick membrane was attached to a 4 mm thick carrier PDMS, and the entire structure was mounted on the oxygen sensing plate. Cell culturing: **b** the microfluidic channel structure sealed with the 20 µm thick membrane was combined with a previously published open-compartment coculture device that had three compartments connected to each other with microtunnels that had dimensions of 10 µm width, 3.5 µm height and 250 µm length (Ristola et al. 2019)

The gas channel inlets and outlets were punched with a modified 18G needle. The microfluidic channel structure was closed with a 20 µm Silpuran membrane. For oxygen characterization purposes, the sealed microfluidic channel structure was bonded to a 4 mm thick carrier PDMS (1:10) piece and to the oxygen sensing plate such that the 20 µm thick membrane faced the oxygen sensing plate (Fig. 2a). All the parts were bonded with oxygen plasma (Pico, Diener Electronic GmbH + Co. KG, Ebhausen, Germany) for 20 s (PDMS parts) or 15 s (PDMS parts to the oxygen sensing plate) at 30 W and 0.3 mbar pressure. After fabrication, gas flow rates were measured from each outlet separately to confirm the functionality of the channels.

In the cell culture experiments, the sealed microfluidic channel structure was bonded with the previously published open-compartment coculture device, which had three compartments connected to each other with microtunnels that had dimensions of 10 µm width, 3.5 µm height and 250 µm length (Ristola et al. 2019). The three-compartment device was placed atop the 20 µm membrane of the microfluidic channel structure so that the cells were cultured on top of this membrane (Fig. 2b). The whole structure was bonded to a glass plate. We have evaluated with COMSOL Multiphysics (Version 6.0 COMSOL, Inc., Burlington, USA) simulations that the presence of microtunnels between the cell compartments have a minimal effect on the generated oxygen environments (See Supplementary Material Fig. S3 and Fig. S4).

### Mold characterization and physical dimensions of the channels

The microfluidic channel height in the SU-8 mold were measured with a contact profilometer (Dektak XT, Bruker, Billerica, MA, USA). The dimensions of the 2 µm microchannel array in the cast PDMS components were characterized using a scanning electron microscope (SEM, ULTRAplus, Carl Zeiss, Oberkochen, Germany). The casted structures were cut perpendicular to the orientation of the microchannel arrays, and the cut pieces were attached to aluminum SEM stubs using carbon tape so that the cross-sections were facing upward. Subsequently, the samples were coated with an approximately 4 nm thick Pt/Pd coating.

### Ratiometric oxygen sensing setup

To characterize the spatiotemporal behavior of the microfluidic channel structure, a ratiometric 2D oxygen imaging system was applied according to Ungerbök et al. (Ungerböck et al. 2013). Briefly, the imaging system employs the red channel of an RGB camera as an oxygen-sensitive channel and the green channel as a reference channel. Compared to the pure intensity-based methods, the ratiometric method provides a means to compensate against many interfering factors, especially against inhomogeneities and fluctuations the illumination field. Platinum(II)-5,10,15,20-tetrakis-(2,3,4,5,6-pentafluorphenyl)-porphyrin (PtTFPP) (Livchem Logistics GmbH, Frankfurt, Germany) was used as the oxygen-sensitive dye, and Macrolex Fluorescent Yellow (MFY) (Livchem Logistics GmbH, Frankfurt, Germany) was used as the reference dye.

The oxygen sensing plates were fabricated by knife coating. Briefly, 40 mg of PtTFPP, 80 mg of MFY and 4.00 g of polystyrene (PS) pellets (Sigma Aldrich, Sain Louis, USA) were dissolved in 35.84 g of chloroform. Glass plates (dimensions of 49 mm × 49 mm × 1 mm from Gerhard Menzel GmbH, Braunschweig, Germany) were sonicated in isopropanol and acetone, rinsed with deionized water, and dried with nitrogen. The plates were treated with oxygen plasma for five minutes and precoated with hexamethyldisilazane (HMDS) in a vacuum at 150 °C (Hotplate HMDS-OPTIhot SVT20, ATMgroup, Marklowice, Poland).

A volume of 400 µL of dye solution was pipetted on the plate, and a knife-coater set to 140 µm was used to spread the solution. The coated plates were left to dry overnight at room temperature. The resulting oxygen sensing film had an approximate thickness of 14 µm, and it contained approximately 1.0% w/w of PtTFPP and 2.0% w/w of MFY in PS. Similar to Ungerböck and coworkers, we also found that a ratio of 1:2 between PtTFPP and MFY worked well (Ungerböck et al. 2013).

A CMOS RGB camera (Canon EOS 80D, Canon Oy, Tokyo, Japan) mounted on a microscope (Zeiss Axio Observer Z1 Carl Zeiss AG, Oberkochen, Germany) was used to acquire images at defined intervals. Images were acquired with a 2.5X objective (Objective "A-Plan" 2.5x/0.06 M27 Carl Zeiss AG, Oberkochen, Germany) and using an FITC filter set consisting of a bandpass 450–490 nm excitation filter, a 510 nm dichroic mirror and a longpass 515 nm emission filter illuminated with a 50 W mercury short arc lamp (OSRAM 50 W HBO 50 W/AC L1 mercury short arc 69,213–1 lamp by Osram GmbH, Berlin, Germany). The acquired raw images were analyzed in MATLAB (version R2021b, The MathWorks Inc, Massachusetts, USA) using a custom-made code for ratiometric measurements. The plotting of the final results was performed with MATLAB code by Musall (2022). The collected images were edited in Photoshop (Adobe Inc. California, USA) to adjust contrast and saturation for improved visibility. The fall times and rise times of the oxygen concentration were calculated with MATLAB built-in functions. Paired samples t-test was used to assess the statistical significance of the results.

### Oxygen measurements

To characterize the spatiotemporal oxygen behavior of the microfluidic channel structure, the microfluidic channel structure was mounted on the top of the oxygen sensing plate, and the ratiometric oxygen sensing setup was employed. With the 2.5X objective, the field of view (FOV) in the camera is approximately 3.56 mm × 5.33 mm, which limits the number and area of the compartments that fit into the FOV. However, the FOV extends to two compartments, which is sufficient for functional characterization due to the fast imaging and symmetry of the device. Both Compartments B1 and B2 were measured at least once, and the averages of these measurements are referred to as Compartment B when applicable. The oxygen measurements were repeated three individual times with one device, including the calibration. Table 1 lists all the gas mixtures used in the calibration and measurements.

**Table 1:** Gas mixtures used in the calibration and measurements

	**Gas mixture**
M19	19% O_2_, 76% N_2_, 5% CO_2_
M5	5% O_2_, 90% N_2_, 5% CO_2_
M1	1% O_2_, 94% N_2_, 5% CO_2_
M0	0% O_2_, 95% N_2_, 5% CO_2_

#### Calibration

The same gas mixtures were supplied sequentially (M19, M0, M5, M1, M19) to all compartments. To ensure the stabilization of the oxygen concentration, each mixture was allowed to flush through the channels for 60 min. Fluorescence images were acquired at 2-min intervals. In MATLAB, the red and green channels were separated, and on each defined region of interest (ROI), the ratiometric parameter value *R* was calculated by dividing the mean value of the red channel by the mean value of the green channel. The ratiometric parameter values of each gas mixture were fitted to the simplified two-site Stern–Volmer equation (Demas et al. 1995):

1 $$\frac{R}{{R}_{0}}=\frac{{f}_{1}}{1+{K}_{sv}\left[{O}_{2}\right]}+{f}_{2}$$

where *R* and *R*_*0*_ are the ratiometric values under an oxygen concentration of [O_2_] and in the absence of oxygen, respectively; *f*_*1*_ represents the fraction of dye molecules, which is quenched with the Stern–Volmer coefficient *K*_sv_ and *f*_*2*_ = *1 - f*_*1*_ represents the nonquenchable fraction of dye molecules. The fitting procedure generated estimates for parameters *f*_*1*_ and *K*_*sv*_, which were used to calculate the oxygen concentration during the measurements. The calibration procedure was carried out before each measurement.

#### Measurement

After completing the calibration phase, all compartments were supplied with an M19 gas mixture for an additional ten minutes. To resolve the surface oxygen profile dynamics, the following measurement procedure was used:

1. M0 gas mixture supplied to all compartments for 30 min
2. M19 gas mixture supplied to all compartments for 30 min, bringing the system to the initial state
3. M0 gas mixture supplied to Compartment A and M19 gas mixture supplied to Compartments B1 and B2 for 30 min
4. M19 gas mixture supplied to all compartments for 30 min, bringing the system to the initial state
5. M19 gas mixture supplied to Compartment A and M0 gas mixture supplied to Compartments B1 and B2 for 30 min

To follow the rapid oxygen dynamics, the fluorescence images were recorded with a five-second interval.

### Cell culture

Mouse embryonic fibroblast (MEF) cells (MEF (CF­1) cells, SCRC­1040, ATCC (American Type Culture Collection), Manassas, USA) were passaged in complete medium containing Dulbecco's modified Eagle’s medium (DMEM), high glucose, pyruvate (Thermo Fisher Scientific, Massachusetts, USA) with 15% fetal bovine serum (FBS) (Sigma Aldrich, Missouri, USA), 100 IU/ml penicillin and 100 µg/ml streptomycin (Sigma Aldrich, Missouri, USA) in T75 culture flasks and cultured in a 37 °C humidified incubator with 5% CO_2_. Cells under passage number ten were used for device experimentation.

The PDMS cell culture devices were treated with oxygen plasma (Pico, Diener Electronic GmbH + Co. KG, Ebhausen, Germany) for 4 min at 50 W and 0.3 mbar, and the cell culture compartments were coated with 50 µg/ml fibronectin (ProtMarket Research infrastructure, Tampere, Finland) dissolved in Dulbecco’s phosphate buffered saline (DPBS). The coating solution was incubated for one hour at 37 °C in a humidified incubator with 5% CO_2_. The coating solution was replaced with complete medium filling half of the chamber volume prior to cell seeding.

The cells were detached with 1 × trypsin (Thermo Fisher Scientific, Massachusetts, USA) and resuspended in fresh complete medium after centrifugation. The cells were seeded into the cell culture compartments on top of the coated PDMS membrane at a density of 40 × 10^3^ cells per cm^2^. Medium was added to the top compartment and was changed every other day. The devices were cultured for ten days in an incubator at 37 °C and 5% CO_2_.

### Cell viability

To evaluate the suitability of the minimally manipulated PDMS surface as a culture substrate for live cells, a viability assay was performed on cultured cells without any gas manipulation. The devices seeded with cells were cultured for three, seven and ten days in a 37 °C incubator with 5% CO_2_. Viability from three individual devices was evaluated after three, seven and ten days with a Live/Dead Viability/Cytotoxicity Kit according to the manufacturer’s instructions (L3224 Invitrogen, L3224; Thermo Fisher Scientific, Massachusetts, USA) using 4 µM ethidium homodimer-1 (EthD-1) and 2 µM calcein-AM. Live and dead cells were imaged with an Olympus IX51 inverted fluorescence microscope (Olympus Corporation, Hamburg, Germany), and images were processed in Fiji ImageJ software (1.53f51, NIH, USA). Nine images from three individual devices were used to calculate the viability of cells in each timepoint using software based on Fiji ImageJ particle analysis by Allevi (Allevi Protocols 2022).

## Results and discussion

The results of the measurement of the gas channel dimensions are provided in the Supplementary material (Table S1 and Fig. S1). Briefly, the results show that a designed channel width of 2 µm, height of 2 µm and spacing of 2 µm were achieved in the microfluidic channel structures (Fig. S1). In the following, the results regarding the functional characterization of the device and the cell culture experiments indicating the functionality and cell culture compatibility are discussed.

### Measurements of oxygen profiles

#### Calibration

**Fig. 3:**
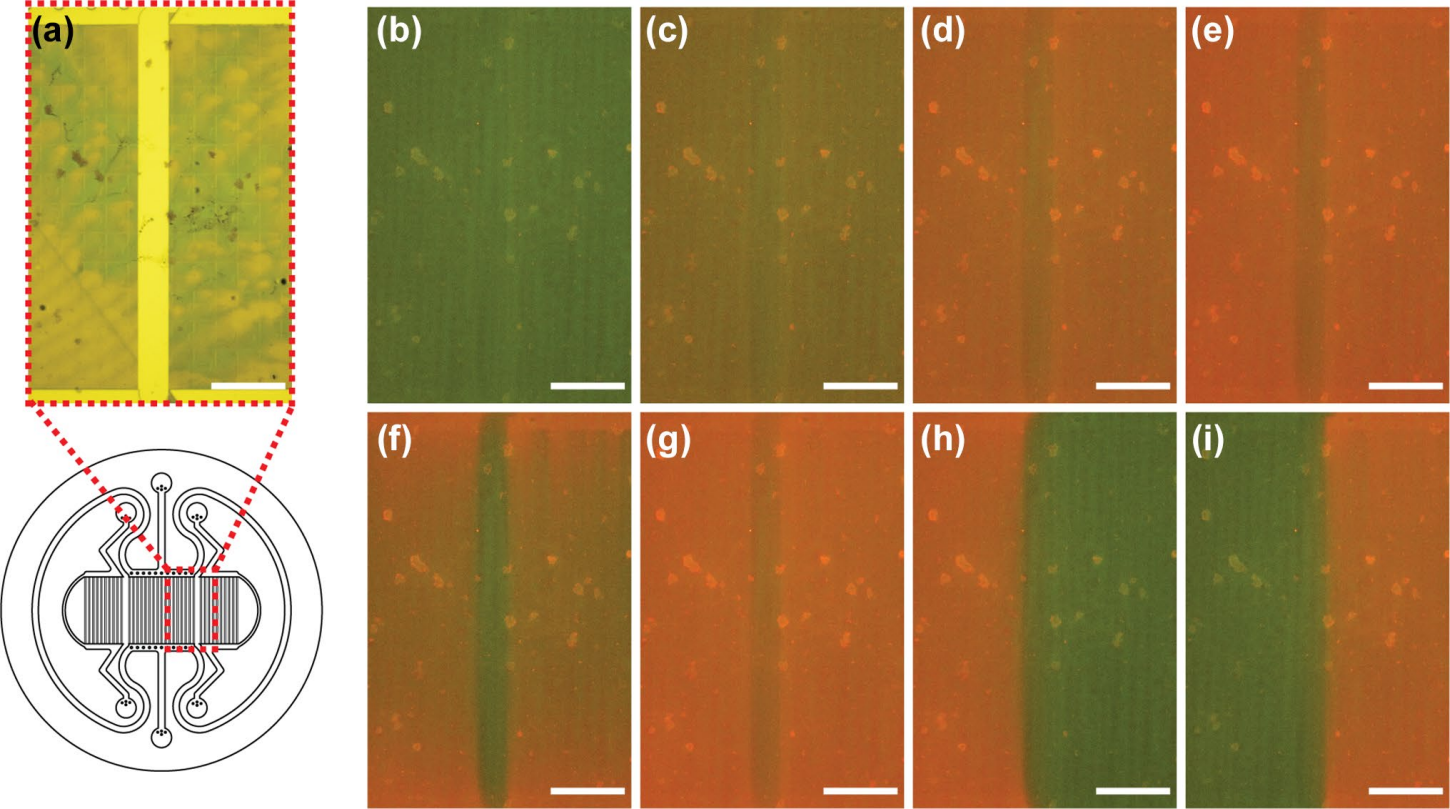
Images of the microfluidic channel structure mounted on an oxygen sensor plate. **a** Brightfield image depicting the measured area and corresponding calibration of the fluorescence images of the same area stabilized to **b** 19%, **c** 5% **d** 1% or **e** 0% oxygen. Measurements show changes **f** after one minute of 0% oxygen. **g** Image taken 30 min after the supply of 0% oxygen showing that the surrounding structures follow the intensity changes of the channels, indicating oxygen diffusion from the bulk PDMS. The array areas in **h** and **i** create distinct oxygen profiles when one is supplied with 19% oxygen and the other with 0% oxygen for 30 min. Scale bar: 1 mm

Figure 3a shows a brightfield image of the measurement area, containing a part of Compartment A and a part of Compartment B2. Figures 3b–e show fluorescence images of the same area under four stabilized oxygen conditions during the calibration. Figure 3b depicts the microchannel arrays at 19% oxygen, and the image appears mainly green, as expected. The brighter spots and the light stripes are artifacts left by the knife coating. When moving from Fig. 3b to Fig. 3e, the red intensity increases as the supplied gas mixture contains less oxygen.

#### Characterization of the device functionality

Figures 3f–i show the fluorescence images of the same area at four distinct time points during the measurement. Figure 3f shows that the red intensity at the microchannel array areas significantly increased one minute after the start of the M0 gas mixture supply, indicating a rapid decrease in the oxygen tension on the membrane surface. Furthermore, images taken after 30 min of M0 supply (Fig. 3g) show that the PDMS barrier between the microchannel arrays followed the same intensity changes, indicating oxygen diffusion from the bulk PDMS structures over time. When Compartment A was supplied with M0 and both Compartments B1 and B2 were supplied with M19 for 30 min, Compartment A exhibited red intensity, while Compartment B2 on the right exhibited green intensity (Fig. 3h). The opposite can be observed when the gases are inversely supplied for 30 min (Fig. 3i). This demonstrates that the microfluidic channel structures can simultaneously induce distinct oxygen profiles in defined areas.

**Fig. 4:**
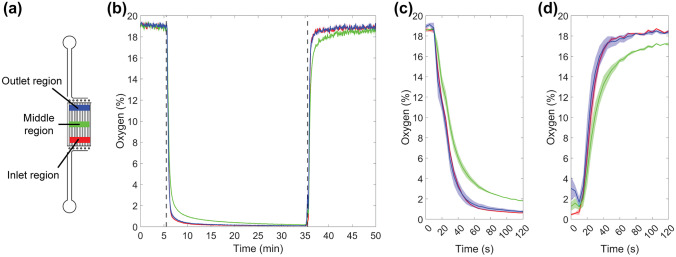
**a** Illustration of the positions where three regions of interest (ROIs) were used to study the Compartment A dynamics. The red ROI indicates the inlet region, the green ROI indicates the middle region and the blue ROI indicates the outlet region. In Fig. 4b-d, the colors of the graphs indicate the region including the mean and the standard error of the mean of the shaded area for n = 3. **b** Graph depicting the surface oxygen tensions of the defined ROIs as a function of time. The dotted lines mark the time t = 5.5 min, where the supplied gas mixture was changed from a gas mixture containing 19% oxygen to 0% oxygen, and the time t = 35.5 min, where the supplied gas mixture was changed from a gas mixture containing 0% oxygen to 19% oxygen. **c** Graph depicting the surface oxygen tensions of the defined ROIs during the step change of the supplied gas mixture containing 19% oxygen to 0% oxygen. **d** Graph depicting the surface oxygen tensions of the defined ROIs during the step change of the supplied gas mixture containing 0% oxygen to 19% oxygen

**Fig. 5:**
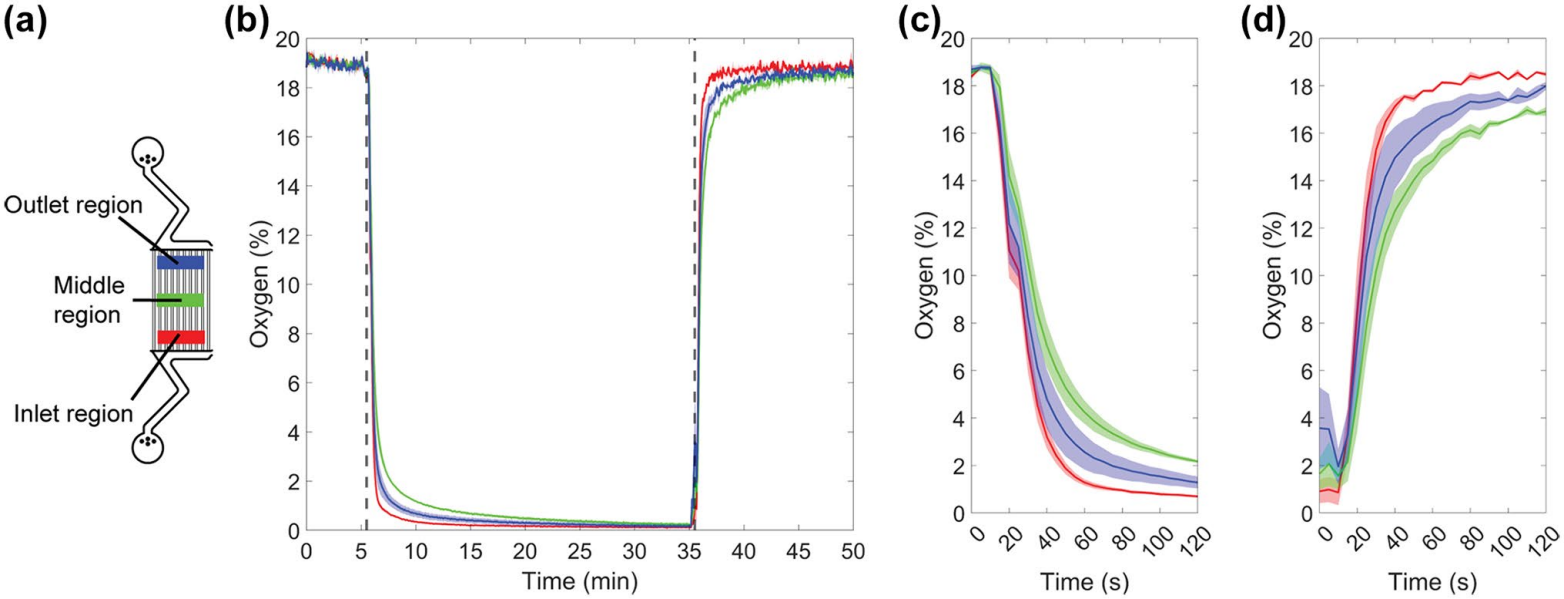
**a** Illustration of the positions where three regions of interest (ROIs) were used to study the Compartment B dynamics. The red ROI indicates the inlet region, the green ROI indicates the middle region and the blue ROI indicates the outlet region. In Fig. 5b-d, the colors of the graphs indicate the region including the mean and the standard error of the mean of the shaded area for n = 3. **b** Graph depicting the surface oxygen tensions of the defined ROIs as a function of time. The dotted lines mark the time t = 5.5 min, where the supplied gas mixture was changed from a gas mixture containing 19% oxygen to 0% oxygen, and the time t = 35.5 min, where the supplied gas mixture was changed from a gas mixture containing 0% oxygen to 19% oxygen. **c** Graph depicting the surface oxygen tensions of the defined ROIs during the step change of the supplied gas mixture containing 19% oxygen to 0% oxygen. **d** Graph depicting the surface oxygen tensions of the defined ROIs to step change of the supplied gas mixture containing 0% oxygen to 19% oxygen

Figures 4 and 5 depict responses to step changes in the gas supply in Compartments A and B, respectively. In both figures, at time t = 5.5 min, the gas mixture is changed from M19 to M0, and at time t = 35.5 min, the supplied mixture is changed from M0 to M19. To study the dynamics at individual locations in detail, individual regions of interest (ROIs) with a size of 600 × 100 pixels were defined in positions illustrated in Figs. 4a and 5a. The fitting procedure according to Eq. 1 was performed for each ROI separately, yielding calibration parameters. According to this, surface oxygen concentrations were resolved for each ROI and plotted as a function of time (Figs. 4 and 5). The results show that in each ROI, the surface oxygen concentration rapidly changed with the condition in the gas channels and that most of the changes took place in under a minute. Interestingly, in both compartments, the regions in the middle showed slightly slower dynamics compared to the regions closer to the inlet and outlet. This could be due to a restricted gas flow in the microchannels and unideal channel fabrication in some part of the structure. In addition, oxygen diffusion from the ambient environment could interfere with the measurement. Similarly, the region at the outlet side of Compartment B showed slower dynamics than the region at the inlet side (Fig. 5). This could be due to the different channel designs of Compartments A and B. Nonetheless, the microfluidic channel structure is capable of rapidly decreasing the surface oxygen concentration to hypoxic levels and reverting it back to normoxic levels. Thus, the device is capable of modeling the hypoxic insult and the subsequent reperfusion phase as well as the cyclic oxygen profiles, where the oxygen tensions fluctuate periodically. The lowest oxygen levels achieved were below 0.1%, and even the middle regions achieved values below 0.3% within the measurement time of 30 min.

**Fig. 6:**
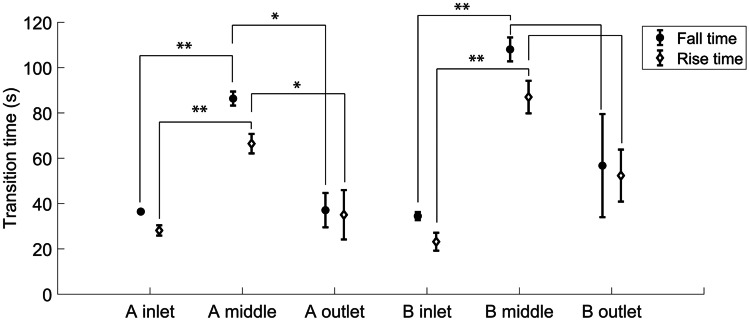
Averages and standard deviations (n = 3) of the calculated fall times and rise times of different ROIs in Compartment A (A inlet, A middle and A outlet) and Compartment B (B inlet, B middle and B outlet). The statistical significances (paired t-test) are denoted as * p < 0.05 and ** p < 0.01

The fall times and the rise times were calculated for each ROI and measurement separately from the 10% and 90% signal levels, and the results are depicted in Fig. 6. The fall time describes the time required to decrease the oxygen concentration from normoxic to hypoxic conditions, and the rise time describes the time required to increase the oxygen concentration from hypoxic to normoxic conditions. The fall times varied between 32 and 114 s, and the rise times varied between 19 and 95 s. In both compartments, the fall times and rise times were longer in the middle region compared to the inlet and outlet regions. In Compartment A, the differences were statistically significant for both the inlet region (p < 0.01) and the outlet region (p < 0.05), while in Compartment B only the inlet region showed a statistically significant difference (p < 0.01). Nevertheless, it remains a further research to study whether decreasing the bulk PDMS volume in the microchannel array would decrease the transition times in the middle regions. The bulk PDMS volume could be decreased by making the gas channels wider than the PDMS walls in the microchannel arrays. This would further increase the surface area towards the 20 µm membrane, thus improving the gas diffusion. We have estimated that due to the dead volumes in the tubing and in the valve system, it took ten seconds for the gases to reach the microfluidic structure. When manually changing the gas mixtures from 0% oxygen to 19% oxygen, the gas flow was stopped, and the pressure released from the tubing and the surrounding air had brief access to the system before the supply of another gas mixture. This possibly aided the faster dynamics that occurred under increasing oxygen concentration. In addition, it is important to take into account that these values were acquired from devices containing no cells. Typically, a cell culture acts as an oxygen sink and the oxygen consumption is dependent on the cell type, the amount of cells and their metabolic state (Oomen et al. 2016). Thus, the spatial oxygen distribution should be evaluated for each cell application separately. Regarding the dynamics, cells as oxygen sinks can significantly accelerate changes toward low oxygen concentrations. Within this system, low oxygen levels were established in under two minutes, even though the diffusion of oxygen from the surrounding air and the bulk PDMS was not prevented in any way as in other published designs (Funamoto et al. 2012; Oppegard et al. 2010). Overall, the channel structure is capable of changing the oxygen tension on a time scale relevant to biological phenomena, such as brain stroke, where the damage is already visible within 2–3 min from the start of the hypoxic insult (Murphy et al. 2008).

**Fig. 7:**
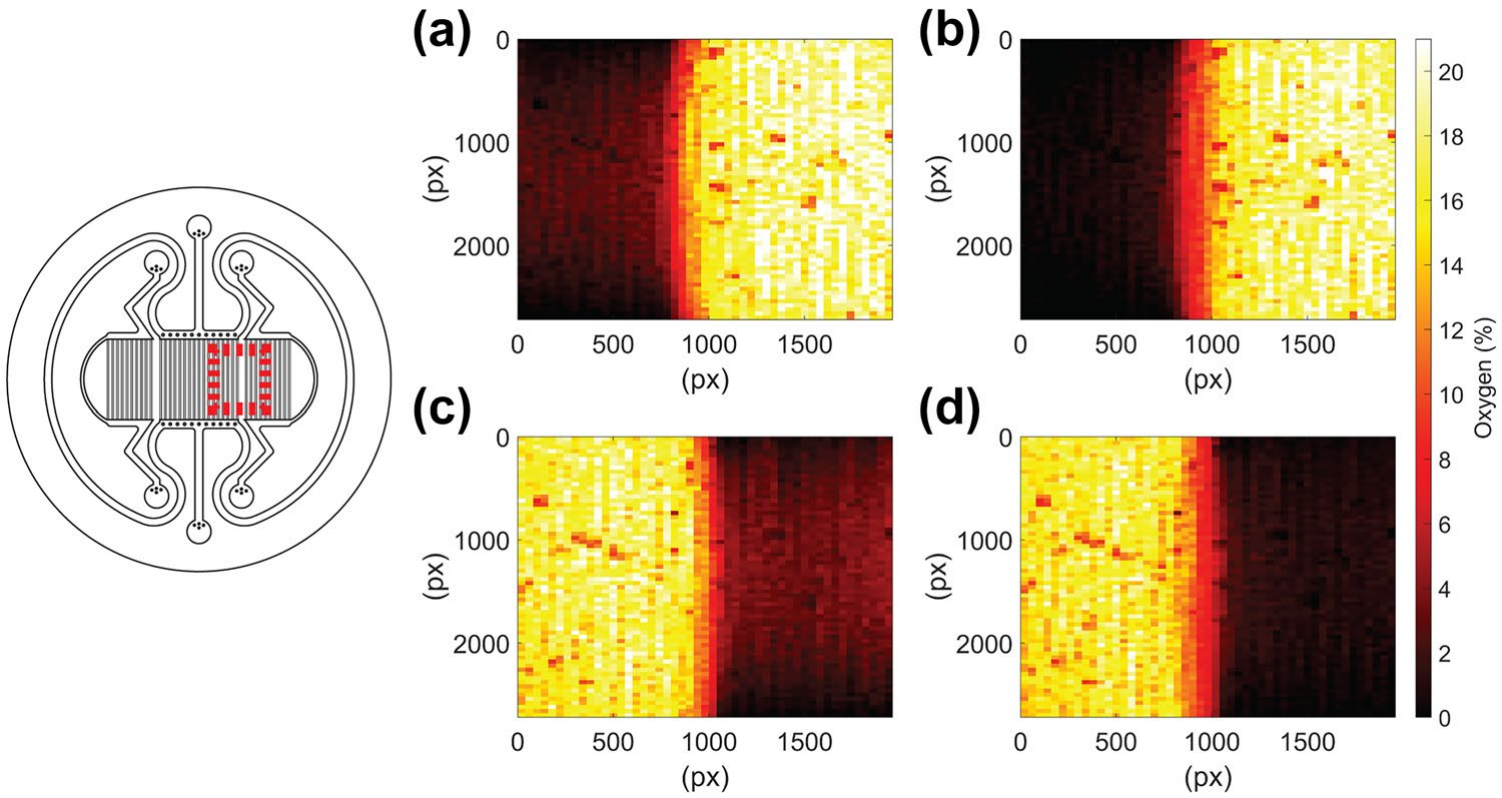
Heatmaps depicting the surface oxygen tensions after **a** 1 min and **b** 30 min of 0% oxygen supply to Compartment A and 19% oxygen supply to Compartment B. The surface oxygen tensions after **c** 1 min and **d** 30 min of 19% oxygen supply to Compartment A and 0% oxygen supply to Compartment B

To study the spatial responses with a better resolution, 2D profiles of the surface oxygen concentration across two of the microfluidic arrays were calculated at different time points. A large ROI (2720 × 1960 pixels) was defined and then divided into a matrix of minor regions (each 40 × 40 pixels). Using the averages of previously estimated calibration parameters for the measurement, the surface oxygen concentrations were calculated at each minor region. These results are plotted as heatmaps in Fig. 7. These results again show that the device can create hypoxic environments in one compartment, while the other compartment maintains oxygen tension within normoxic values. Since the system contains three separately controlled compartments, three different oxygen environments of the hypoxic tissue sites could be created simultaneously in the same platform. Due to the nature of the PDMS material and good oxygen permeability, Fig. 7 shows that neighboring compartments slightly influence each other, creating a region between them where the oxygen concentration does not resemble that of either of the compartments (Fig. 7). The region is generated by the PDMS wall between the compartments. In some cell culture configurations, microtunnels providing a connection between the compartments can be placed on this PDMS wall (Fig. 2b). A typical example could be a compartmentalized neuronal model, where the microtunnels would allow axonal growth and interaction between the compartments (Ristola et al. 2021). The configuration would allow oxygen diffusion through the microtunnels, but as the oxygen diffusion coefficient of liquid and PDMS are of the same order, the effect remains limited (Table S2 and Figs. S3–S4). The compartments themselves are capable of creating distinct oxygen environments despite the microtunnels and the permeable nature of PDMS even in close proximity to each other.

### Cell viability

**Fig. 8:**
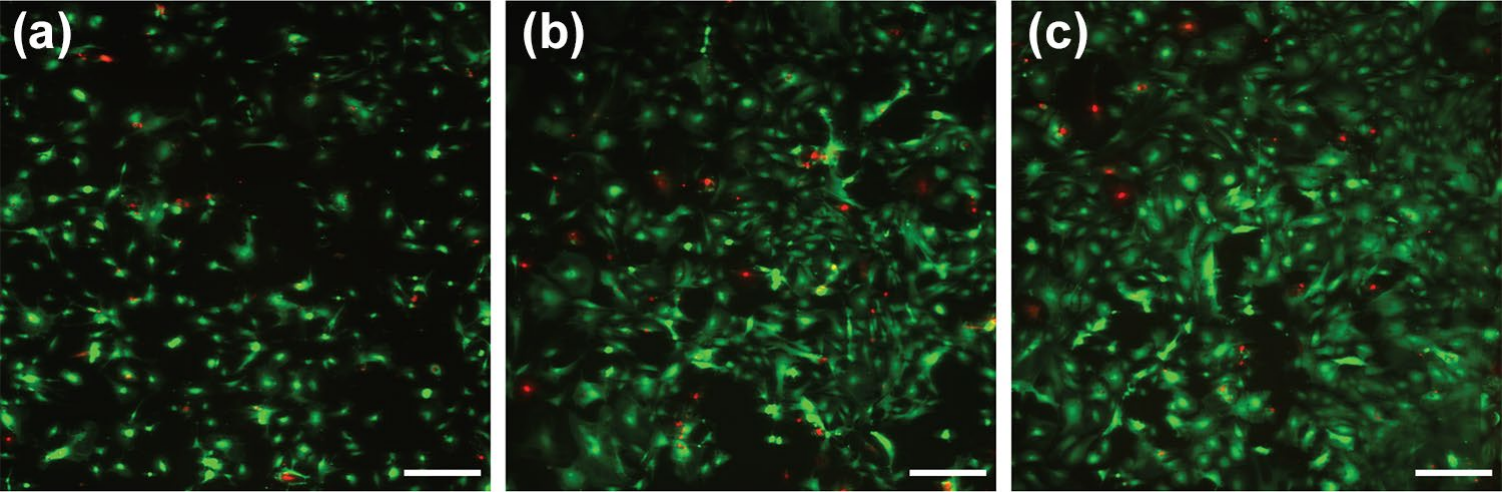
Live/dead staining of MEFs cultured for **a** 3 days, **b** 7 days and **c** 10 days in PDMS devices coated with fibronectin. Living cells are shown in green, and dead cells are shown in red. Scale bar: 400 µm

Mouse embryonic fibroblast cells were plated on microfluidic channel structures coated with fibronectin and cultured for up to ten days in an incubator. Live-dead staining was performed on Days 3, 7 and 10, and the results showed minimal cell death for up to ten days (Figs. 8 and S5). Furthermore, the cells proliferated in the devices. This suggests that the developed structure is suitable for cell culturing. Since the fabricated microchannel array is a periodically modulated surface, it has some diffraction grating properties (Bonod and Neauport 2016) observed as light dispersion affecting the visualization of cells. Nevertheless, fluorescence images of sufficiently high quality for cell viability analysis were acquired. In the cell culture configuration, the microfluidic channel structure is positioned under open well structures (Fig. 2b), meaning that oxygen has access to the culture medium from the top of the device. However, Rexius-Hall et al. showed that when gases with low oxygen concentrations are supplied beneath the cells through a 100 µm PDMS membrane, the oxygen conditions within 100 µm of the membrane can be maintained as low despite the upper parts being exposed to the surroundings (Rexius-Hall et al. 2014). We propose that with cell type-specific coatings, the microfluidic channel structure is a suitable substrate for cell culture, and by combining the microfluidic channel structure with the coculture compartments, oxygen responses of different cell types can be studied using this platform.

## Conclusions

In this study, we presented a microchannel structure for the spatiotemporal control of oxygen microenvironments. The structural dimensions, oxygen modulation dynamics and spatial resolution for oxygen environment control were characterized. The structure was capable of creating a large variety of oxygen profiles between 19 and 0%, and the oxygen environment was modulated within two minutes. The oxygen modulation dynamics represent a time scale that is relevant for pathologies, such as stroke. In addition, the structure can simultaneously create distinct oxygen profiles in defined cell culture compartments that minimally interfere with each other. We have shown that the microfluidic channel structure is a suitable cell culture substrate with a cell type-dependent surface modification. Combining the structure with a coculture device, different oxygen microenvironments can be established in separate compartments, while cells stay connected via microtunnels. This device could be utilized, for example, in stroke or cancer research, where precisely defined and controlled oxygen environments will provide more *in vivo*-like environments for studying cell responses.

## Supplementary Information

Below is the link to the electronic supplementary material.

Supplementary file1 (PDF 450 KB)
