# Supplementary material for: Compartmentalized organ-on-a-chip structure for spatiotemporal control of oxygen microenvironments
Source: Biomed Microdevices. 2022 Oct 21;24(4):34. doi: 10.1007/s10544-022-00634-y (PMC9587069; doi:10.1007/s10544-022-00634-y)
Supplement: Supplementary file 1 — Supplementary file1 (PDF 450 KB) [file 10544_2022_634_MOESM1_ESM.pdf]

Compartmentalized organ-on-a-chip structure for spatiotemporal control of oxygen microenvironments

Biomedical Microdevices

Tornberg K.[1], Välimäki H. [1], Valaskivi S. [1], Mäki A-J. [1], Jokinen M. [1], Kreutzer J. [1], Kallio P. [1]

[1] Micro- and Nanosystems Research Group, Faculty of Medicine and Health Technology, Tampere University, 33720 Tampere, Finland

E-mail of the corresponding author: kaisa.tornberg@tuni.fi

Supplementary material

This supplementary material presents the height characterization results from the SU-8 mold (Table S1) and dimension characterization from the casted PDMS microchannel arrays (Fig. S1). Along with that, numerical model was developed to evaluate the effect of microtunnels between two cell culture compartments (see Section Numerical simulations). Finally, results of quantification of Live/dead stained mouse embryonic fibroblast (MEF) cells cultured for three, seven and ten days in polydimethylsiloxane (PDMS) devices coated with fibronectin are presented in Figure S5.

Structural dimension characterization

Table S1 Microfluidic channel heights in the multilayer SU-8 mold measured with a contact profilometer. Four different positions from the mold were measured (n = 4), with the table representing the averages and standard deviations of these measurements.

	**Average height (µm)**	**Standard deviation (µm)**
Microchannel array	2.14	0.07
Inlet and outlet channels together with bypass channels	104.59	0.69

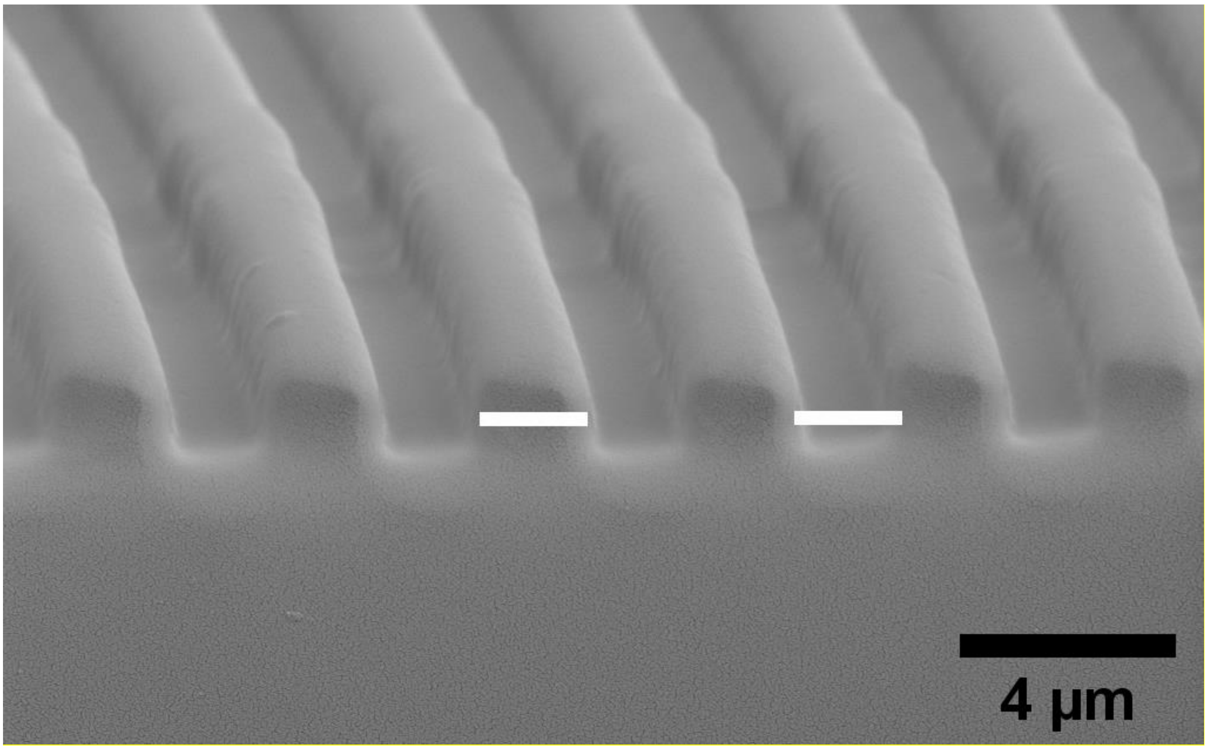

Fig. S1 PDMS microchannel array and (a) acquired SEM image of the 2 µm wide channels with 2 µm spacing (indicated as white bars)

Numerical simulations

In cell culture experiments, the developed microfluidic gas channel structure is attached below an open-compartment coculture device (see Fig. 2b). The device has three compartments connected to each other with microtunnels (Ristola et al. 2019). In cell culture experiments, the compartments are filled with cell culture medium and the cells grow at the bottom of the compartments on a 20-µm thick Silpuran membrane. The microfluidic gas channel structure is located below the membrane. To study how the microtunnels between the compartments affect oxygen profiles in each compartment, we developed a 2D finite-element model using COMSOL Multiphysics (Version 6.0 COMSOL, Inc., Burlington, USA). In the simulations, we applied *The Transport of Diluted Species* interface and used Fick's law as the governing equation to calculate the oxygen concentration profiles with and without the microtunnel structure.

In the device, oxygen is stored in gas, liquid, and solid (PDMS in this case) phases. For this, mass balance, oxygen concentration, and flux in the three phases (gas, liquid, and PDMS denoted with g, l and p, respectively) are calculated using the following equations. Similar modeling approach has been used earlier to model carbon dioxide concentrations and fluxes (Mäki et al., 2015; Mäki 2018). By assuming no oxygen consumption nor

convection in the system, the following mass transportation equations based purely on diffusion can be used for describing a mass balance in the system

$$\frac{\partial c_g}{\partial t} + \nabla\left(-D_g \nabla c_g\right) = 0$$
$$\frac{\partial c_l}{\partial t} + \nabla\left(-D_l \nabla c_l\right) = 0 \qquad S1$$
$$\frac{\partial c_p}{\partial t} + \nabla\left(-D_p \nabla c_p\right) = 0$$

where c and D are the concentrations and diffusion coefficients of oxygen in different phases.

Between the interfaces of the three phases, the rate constants of oxygen mass transport are denoted as follows.

$$c_g \underset{k_{lg}}{\overset{k_{gl}}{\leftrightharpoons}} c_l, \quad c_g \underset{k_{pg}}{\overset{k_{gp}}{\leftrightharpoons}} c_p, \quad c_l \underset{k_{pl}}{\overset{k_{lp}}{\leftrightharpoons}} c_p \qquad S2$$

where subscripts define source and destination phases; for example, gl refers to the rate constant from gas phase to liquid phase, whereas lg refers the opposite direction.

In a steady state, the dimensionless partition coefficients between two phases, Kp, are calculated using the saturated oxygen concentration values in each phase (Shiku et al., 2006; Skolimowski et al., 2010)

$$Kp_{lg} = \frac{k_{lg}}{k_{gl}} = \frac{c_{gsat}}{c_{lsat}}, \quad Kp_{pg} = \frac{k_{pg}}{k_{gp}} = \frac{c_{gsat}}{c_{psat}}, \quad Kp_{pl} = \frac{k_{pl}}{k_{lp}} = \frac{c_{lsat}}{c_{psat}} \qquad S3$$

Oxygen fluxes Fl between two phases can be described as (Shiku et al., 2006; Skolimowski et al., 2010)

$$Fl_{lg} = k_{lg}c_l - k_{gl}c_g = k_{gl}\left(Kp_{lg}c_l - c_g\right)$$
$$Fl_{pg} = k_{pg}c_p - k_{gp}c_g = k_{gp}\left(Kp_{pg}c_p - c_g\right) \qquad S4$$
$$Fl_{pl} = k_{pl}c_p - k_{lp}c_p = k_{lp}\left(Kp_{pl}c_p - c_l\right)$$

Simulations with and without microtunnels were performed using parameters presented in Table S2. The results, presented in the following Figures S2-S4, indicate that microtunnels are only minimally affecting oxygen concentrations.

Table S2 Used simulation parameters.

Symbol	Definition	Value
D_g	Diffusion coefficient, oxygen in gas (m^2/s)	2.5×10^{-5}
D_l	Diffusion coefficient, oxygen in liquid (m^2/s)	3.5×10^{-9}
D_p	Diffusion coefficient, oxygen in PDMS (m^2/s)	3.5×10^{-9}
k_{gl}	Oxygen mass transport constant between gas and liquid (m/s)	2×10^{-5}
k_{gp}	Oxygen mass transport constant between gas and PDMS (m/s)	2×10^{-5}
k_{lp}	Oxygen mass transport constant between liquid and PDMS (m/s)	1.55×10^{-2}
Kp_{lg}	Dimensionless partition coefficient between liquid and gas phases	37.7
Kp_{pg}	Dimensionless partition coefficient between PDMS and gas phases	4.86
Kp_{pl}	Dimensionless partition coefficient between PDMS and liquid phases	0.13
c_{gsat}	Saturated oxygen concentration in gas phase (mol/m^3)	7.47
c_{lsat}	Saturated oxygen concentration in liquid phase (mol/m^3)	0.20
c_{psat}	Saturated oxygen concentration in PDMS phase (mol/m^3)	1.53

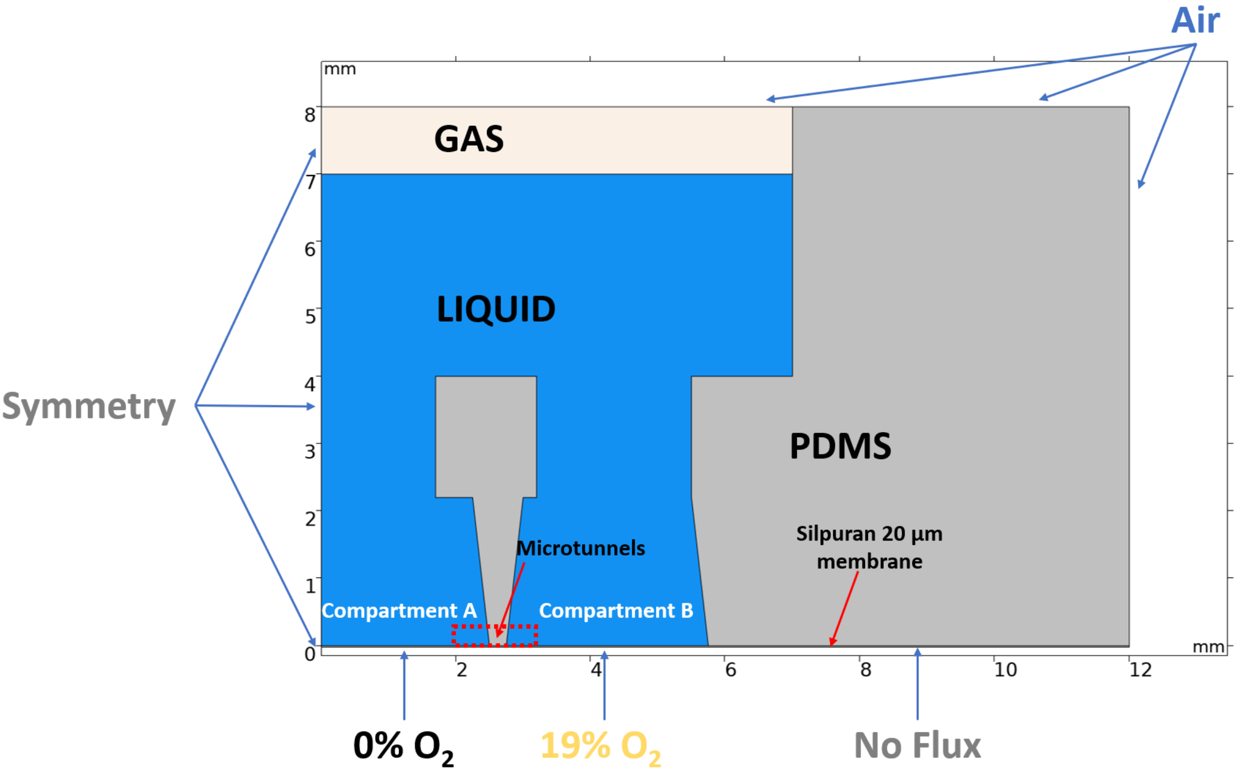

Fig. S2 A 2D symmetrical model of the open-compartment coculture device presented in Fig. 2b. Area marked with the red rectangle is presented in Figure S3. Blue lines indicate boundary conditions applied in the model. In the simulation, 19% oxygen was applied to the microfluidic gas channel below Compartment B and 0% oxygen below Compartment A, water is used as the liquid, ambient air as the gas and PDMS as the solid

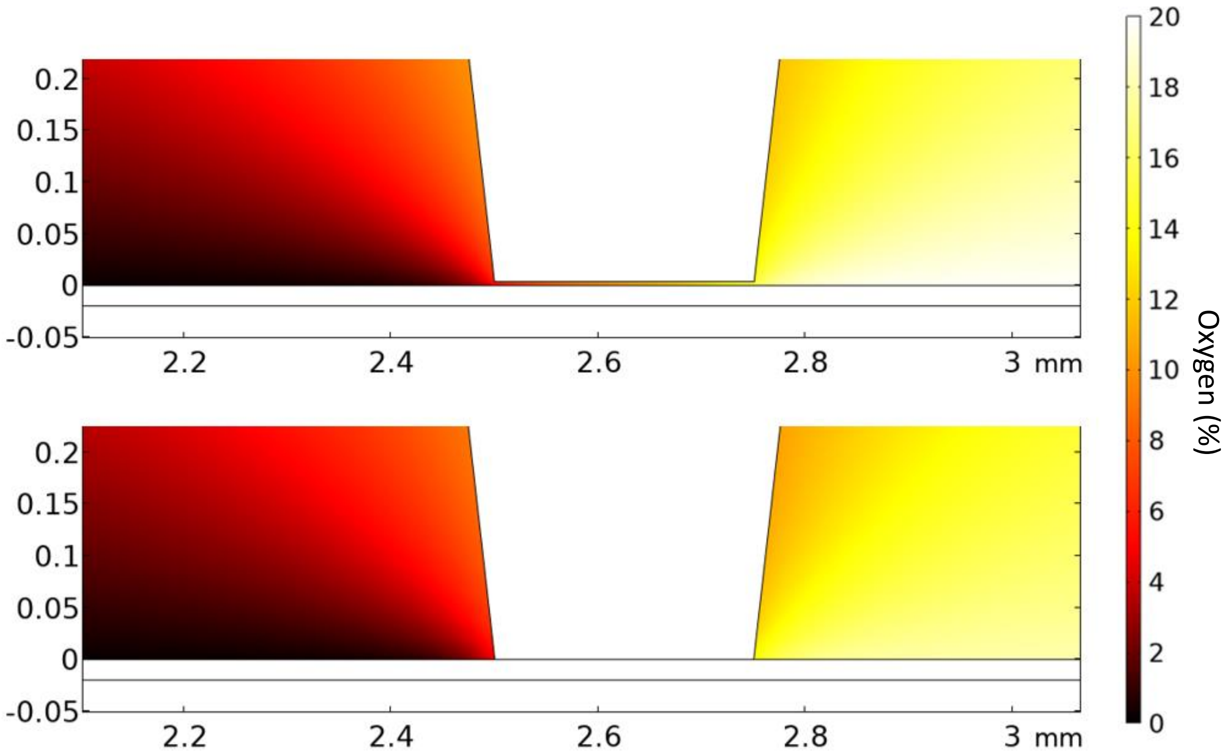

Fig. S3 Simulated oxygen concentration profiles (in the red area shown in Figure S2) with microtunnels (upper figure) and without microtunnels (lower figure) show that the presence of microtunnels between the compartments have minimal effect on the generated oxygen environments

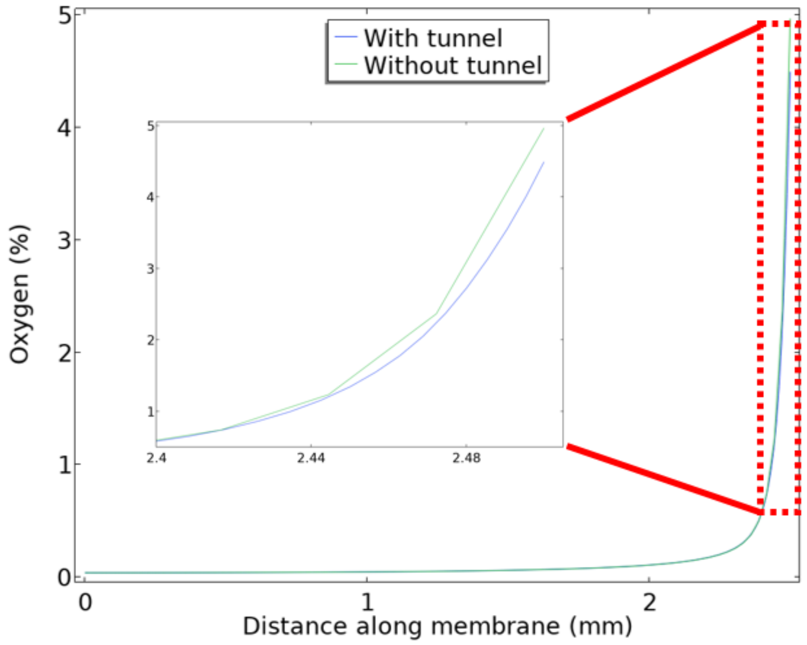

Fig. S4 Figure depicting simulated oxygen concentrations along the membrane in Compartment A with microtunnels (blue line) and without microtunnels (green line) show that the effect of microtunnels is almost negligible

Quantification of Live/dead staining's

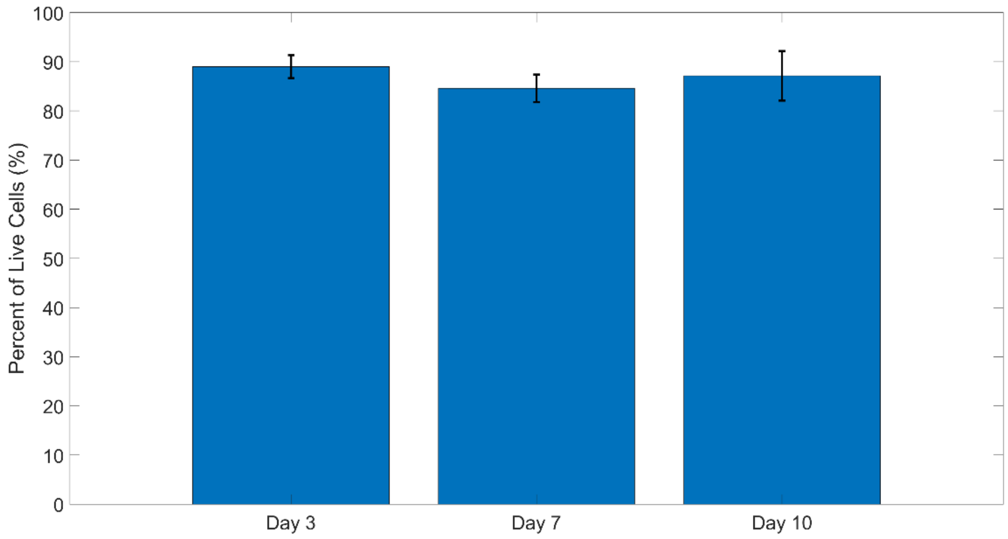

Fig. S5 Quantitative analysis of the percent of live MEF cells cultured in PDMS devices coated with fibronectin for 3 ,7 and 10 days. Cell viability was between 85 – 89 % for the whole 10-day culture period. Bars represent the mean percentage of the live cells in the devices evaluated in each time point, while error bars depict standard deviation (n = 9)

References

Mäki, A.-J., Peltokangas, M., Kreutzer, J., Auvinen, S., & Kallio, P. Modeling carbon dioxide transport in PDMS-based microfluidic cell culture devices. Chemical Engineering Science, **137**, 515–524 (2015). https://doi.org/10.1016/j.ces.2015.06.065

Mäki, A.-J. (2018). Modeling and Control of Microscale Cell Culture Environments (Vol. **1557**) [Tampere University of Technology]. http://urn.fi/URN:ISBN:978-952-15-4174-2

Ristola, M., Sukki, L., Azevedo, M. M., Seixas, A. I., Relvas, J. B., Narkilahti, S., & Kallio P. A compartmentalized neuron-oligodendrocyte co-culture device for myelin research: design, fabrication and functionality testing. Micromechanics Microengineering, **29**(6), 065009 (2019). https://doi.org/10.1088/1361-6439/ab16a7

Skolimowski, M., Nielsen, M. W., Emnéus, J., Molin, S., Taboryski, R., Sternberg, C., Dufva, M., & Geschke, O. Microfluidic dissolved oxygen gradient generator biochip as a useful tool in bacterial biofilm studies. Lab on a Chip, **10**(16), 2162–2169 (2010). https://doi.org/10.1039/c003558k

Shiku, H., Saito, T., Wu, C.-C., Yasukawa, T., Yokoo, M., Abe, H., Matsue, T., & Yamada, H. Oxygen Permeability of Surface-modified Poly(dimethylsiloxane) Characterized by Scanning Electrochemical Microscopy. Chemistry Letters, **35**(2), 234–235 (2006). https://doi.org/10.1246/cl.2006.234
